# SMIM1 at a glance; discovery, genetic basis, recent progress and perspectives

**DOI:** 10.1016/j.parepi.2019.e00101

**Published:** 2019-03-06

**Authors:** Yaw Aniweh, Prince B. Nyarko, Evelyn Quansah, Laty Gaye Thiam, Gordon A. Awandare

**Affiliations:** a West Africa Centre for Cell Biology of Infectious Pathogens, University of Ghana, Accra, Ghana; b Department of Biochemistry, Cell and Molecular Biology, College of Basic and Applied Sciences, University of Ghana, Accra, Ghana

**Keywords:** Vel, Blood, Nucleotides, Frameshift, Heterozygous

## Abstract

Recent elucidation of the genetic basis of the Vel blood group system has offered the field of blood transfusion medicine an additional consideration in determining the causes of hemolytic reactions after a patient is transfused. The identification of the SMIM1 gene to be responsible for the Vel blood group allows molecular based tools to be developed to further dissect the function of this antigen. Genetic signatures such as the homozygous 17 bp deletion and the heterozygous 17 bp deletion in combination with other single nucleotide polymorphisms (SNPs) and insertion sequences regulate the expression level of the gene. With this knowledge, it is now possible to study this antigen in-depth.

## Highlights

• Vel negative phenotype is rare.
• Vel antibodies can cause both mild and severe hemolytic diseases.
• SMIM1 is responsible for the Vel blood group.
• SMIM1 undergoes differential phosphorylation at the cytoplasmic tail.

## 1 The Vel blood group

The Vel blood group was first described in 1952 when a patient had a hemolytic reaction after a blood transfusion (Sussman and Miller, 1952; Storry and Peyrard, 2017). It was discovered that, except for her own blood, Mrs. Vel produced alloantibodies against majority of donors, with only four out ten thousand donor bloods being compatible with hers (Sussman, 1962; Storry and Peyrard, 2017). This phenomenon was later observed in other transfusion procedures as well as in pregnant women, phenocopying Rh reactivity (Daniels, 2013). Some Vel negative (Vel−) individuals produce potent alloantibodies when sensitized either through transfusion with Vel positive (Vel+) blood or during pregnancy with a Vel+ fetus. In rare cases however, individuals produce anti-Vel auto-antibodies (Szalóky and Van Der Hart, 1971; Herron et al., 1979). Anti-Vel antibodies have been shown to robustly lyse Vel+ erythrocytes by activating the complement system (Levine et al., 1955; Szalóky and Van Der Hart, 1971; Herron et al., 1979). In the anti-Vel auto-antibodies instances, both patients were found to suffer from anemia. These observations highlight the importance of Vel blood group in clinical practices, and in hematological studies and applications, as well as the physiological implications of the Vel− phenotype.

## 2 The genetic basis of the Vel blood group

**Fig. 1:**
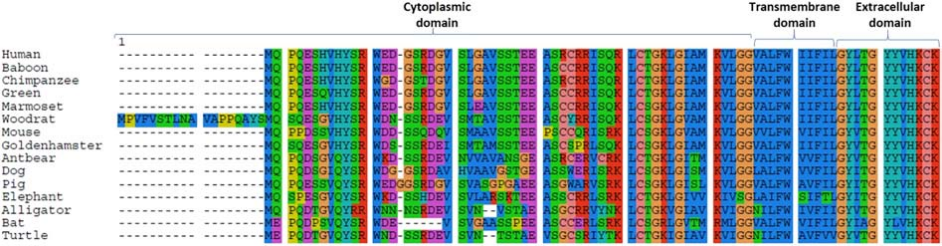
Alignment of SMIM1 amino acids sequences from different animals. It shows a conserved extracellular and transmembrane domain with variable cytoplasmic domain. The colour denotes amino acids with similar properties.

Until recently, the erythrocyte surface antigen responsible for the Vel blood group remained elusive to molecular identification. Using genomics, molecular and biochemical techniques, three research groups reported Small Integral Membrane Protein 1 (SMIM1) to be the Vel blood group antigen (Ballif et al., 2013; Cvejic et al., 2013; Storry et al., 2013). The gene (*SMIM1*) is located within the subtelomeric region of chromosome 1p36 within a 97 kb haplotype block. Interestingly, *SMIM1* is near the Rh gene (Ballif et al., 2013; Storry et al., 2013), and is conserved among members of the animal kingdom (Fig. 1).

The SMIM1 gene consists of 4 exons and 3 introns. However, only exons 3 and 4 code for the functional protein, with exons 1 and 2 untranslated (Ballif et al., 2013). Evidence presently available shows that a homozygous 17 base pair frameshift deletion (64_80del) in exon 3 results in a truncation during mRNA translation and is responsible for lack of the SMIM1 in Vel negative (Vel−) individuals (Ballif et al., 2013; Cvejic et al., 2013; Storry et al., 2013). In addition, a single nucleotide polymorphism (SNP) rs1175550 (A to G substitution) within intron 2 of *SMIM1* has been associated with differential expression level of the gene, with the common A allele correlating positively with reduced protein expression and lower hemoglobin concentrations relative to the G allele, possibly due to the A allele having higher affinity for repressive nuclear proteins (Fehrmann et al., 2011; Van Der Harst et al., 2012; Cvejic et al., 2013; Haer-Wigman et al., 2015a, Haer-Wigman et al., 2015b; Christophersen et al., 2017). Recently, Christophersen and colleagues showed that the G allele of rs1175550 is associated with the specific binding of the transcription factor TAL1, thus providing a possible mechanism for the increased *SMIM1* expression associated with the G allele (Christophersen et al., 2017). In the same study, they identified a new genetic signature rs143702418 (a C to CGCA insertion) which also modulates *SMIM1* expression independent of rs1175550, with the GCA insertion allele accounting for lower expression. Interestingly, there was a linkage between the rs1175550G and rs143702418CGCA as well as the rs1175550A and rs143702418C genotypes in European populations (Table 1). No linkage was however observed in African American populations (Christophersen et al., 2017), suggesting a possible difference in the regulation of *SMIM1*expression between Caucasians and African Americans, and by extension, Africans. In their cohort, the frequency of the alleles rs1175550G and rs143702418C (associated with higher *SMIM1* expression) was 0.26 in the African American population compared to 0.1 in Europeans (Christophersen et al., 2017). Similarly, the frequency of the 17 bp deletion has been shown to be lower in Africans (0.56%) compare to Caucasians (1.46%) (Haer-Wigman et al., 2015a, Haer-Wigman et al., 2015b). These data suggest higher expression of SMIM1 and lower prevalence of Vel negative individuals within the African American population compared to Caucasians. However, the sample sizes, particularly for Africa, used in these studies are inadequate and not representative of the heterogeneous African population. Current data on SMIM1 genetics in sub-Saharan African populations are lacking, thus the need to expand studies to capture this diverse population.

**Table 1:** *SMIM1* alleles and their effect on gene expression.

SNP	Major/minor allele	Effects on *SMIM1* expression
rs1184341	C/T	No
rs2797432	G/A	No
rs143702418	C/CGCA	Yes
rs1181893	C/A	No
rs6673829	G/A	No
rs1175550	A/G	Yes
rs9424296	C/A	No
rs1175549	A/C	No

## 3 The SMIM1 protein

**Fig. 2:**
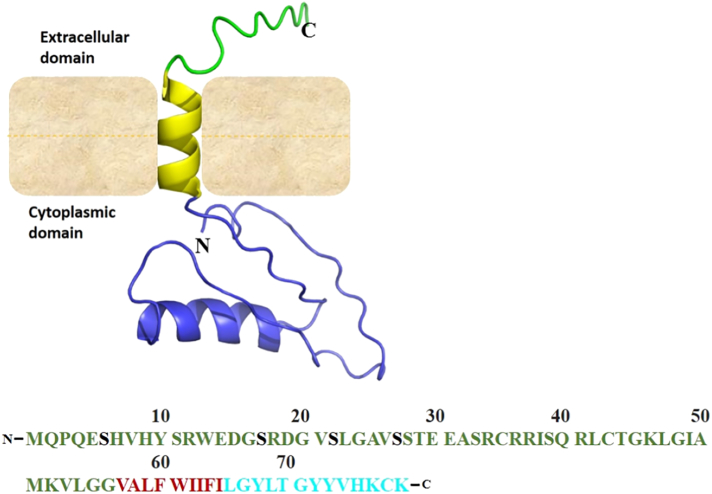
SMIM1 protein. a. The schematic of the SMIM1 protein with I-TASSER predicted 3D-structure showing the extracellular domain (green), transmembrane helix (yellow) and the cytosolic domain (blue) b. Amino acids sequence of the human SMIM1 showing the confirmed different phosphorylation sites (Black) amidst the cytosolic domain (green), transmembrane domain (red) and the extracellular domain (cyan).

*SMIM1* encodes a 78-amino acid, single transmembrane domain (TMD) containing protein with a predicted TMD at amino acids 47 to 67. The protein was previously predicted to be a type I transmembrane protein (Storry et al., 2013) but has recently been shown to be a type II transmembrane domain protein with amino acids at positions 1 to 47 predicted to constitute the cytoplasmic domain while positions 68 to 78 form the extracellular stalk (Fig. 2a, b) (Arnaud et al., 2015).

SMIM1 is highly expressed in hematopoietic cells yet, the exact mechanism of its involvement in erythroid development is limited (Storry et al., 2013). The protein has been shown to undergo posttranslational modification through phosphorylation of serine residues at positions 6, 17, 22 and 27 (Arnaud et al., 2015) (Fig. 2c). As a protein, SMIM1 is conserved in primates. The degree of conservation of the extracellular domain is very high (approximately 100%), followed by the transmembrane domain (98%) and variable cytoplasmic tail (approximately 76%). It is however not clear what interactions this protein engages in at the interface of the cytoskeleton, in the cytosol or during cell-cell adhesion/communication. Additionally, the molecular basis of Vel is not entirely known. It is unclear whether the blood group is determined by the extracellular domain of SMIM1 or post-translational modifications on the protein surface. Thus, this gap in knowledge needs resolution.

## 4 Vel blood group and health

Vel is a high frequency blood group antigen with a negativity occurrence rate of approximately 0.025% in Caucasians (Cedergren et al., 1976; Herron et al., 1979; Daniels, 2013) similar to 0.021% in Brazil (Dezan et al., 2016). To date, available data suggest the prevalence of the Vel negative (Vel−) phenotype is highest in Caucasians, especially in the Scandinavia, while black Africans from Ethiopia, South Africa as well as black Americans show the lowest prevalence (Storry et al., 2013; Haer-Wigman et al., 2015a, Haer-Wigman et al., 2015b). With these data, it is clear that Vel− blood is difficult to obtain for the purpose of transfusion in many countries. The erythrocyte hemolytic activity induced by the anti-Vel response has been described as very aggressive (Sussman and Miller, 1952; Le Masne et al., 1992; Vucinovic et al., 2004; Van Gammeren et al., 2008). In neonates, three different reports have shown that anti-Vel is able to cause mild to severe neonatal hemolytic disease (Le Masne et al., 1992; Vucinovic et al., 2004; Van Gammeren et al., 2008). Despite the breakthrough on the underlying genetics of the Vel blood group, data on *SMIM1* genetics is still limited. Presently, there are no data available for natives of Sub-Saharan Africa.

Currently, no pathophysiological condition has been associated with the lack of SMIM1 in humans, largely attributable to the lack of the physiological function of the antigen. However experiments in zebra fish associated the absence of the protein to low hemoglobin levels (Cvejic et al., 2013). Given that the absence of some blood group antigens results in pathologies ranging from mild to life threatening conditions (Mohandas and Narla, 2005; Amjadi et al., 2015), it is worthwhile to comprehensively identify and classify all possible genetic signatures that account for non-expression or varied expression of SMIM1 in heterogeneous populations. In addition, based on data from the zebra fish experiment, it is logical to investigate any possible pathophysiological condition associated with the lack of SMIM1.

## 5 Typing the Vel blood group

Pre-sensitized Vel− patients are at risk of suffering acute hemolytic reaction should they be mistakenly transfused with Vel positive blood. Similarly, sensitized pregnant women risk losing a Vel+ fetus through hemolytic diseases of the fetus and newborn. These clinical implications of sensitization in Vel− individuals highlight the need to properly identify the Vel status of primigravida women as well as both donor blood and the corresponding recipients. However, there is wide variation in the levels of expression of SMIM1, especially in individuals who are heterozygous for the 17 bp deletion (Ballif et al., 2013; Cvejic et al., 2013; Storry et al., 2013). Furthermore, before the identification of *SMIM1* as expressing the Vel antigen, serology was the only means of typing Vel. However, this approach was quite cumbersome and in some cases unreliable largely because (a) anti-Vel antibodies could only be obtained from the serum of sensitized Vel− individuals, and (b) the expression of the Vel antigen varies significantly among individuals, ranging from high, low (weak) to no expression. Thus, there was the risk of typing a weak expresser as negative.

The varied expressivity of Vel (SMIM1), together with scarcity of antibodies to the antigen made serological typing unreliable. However, identification of the genetic basis of the Vel blood group has led to the development of genetic techniques for typing the Vel antigen (Ballif et al., 2013; Wieckhusen et al., 2015; Costa et al., 2016; Dezan et al., 2016). Additionally, the successful production of anti-Vel monoclonal antibodies (Danger et al., 2016) provides a reliable source of reagents for antibody-based molecular analysis. Another method that was recently used in typing Vel and the different genotypes at rs1175550 (A/G) among Thai donors employed Matrix-Assisted Laser Desorption Ionization coupled Time-of-flight mass spectrometry (MALDI-TOF MS) (Jongruamklang et al., 2018). Much as this method is good, it is limited by the cost as well as proximity to the blood group typing centers. In this study, the investigators identified the variable expression of SMIM1 (Vel) in positive individuals which points to the inaccuracy of serology for distinguishing low SMIM1 (Vel) expressing individuals from negatives (Chandanayingyong et al., 1967). Such variations in SMIM1 expression can easily be determined using flow cytometry (Dezan et al., 2016). The allele-specific and restriction fragment length polymorphism (RFLP) PCR-based methods are robust and can be applied in high throughput for the successful characterization of the Vel genotypes, thus, making Vel typing in both clinical practice and research easy and reliable. However, there are currently no commercially available Vel genotyping platforms for use outside reference/research laboratories, thus making this technology less accessible for clinical application.

## 6 SMIM1 in the *Plasmodium falciparum* infection

The malaria parasite *Plasmodium*, is among a limited number of disease-causing agents that reside in human erythrocytes (Rios and Bianca, 2000). Various aspects of parasite development and disease pathology have been attributed to blood group antigens. For instance, *Plasmodium vivax* requires the Duffy antigen to successfully invade reticulocytes; *P. falciparum* requires the sialic acid moieties on glycophorins, in addition to other receptors such as complement receptor 1 (CR1), decay accelerating factor (DAF), basigin, and band-3, for entry into host erythrocytes (Egan, 2018). Disease phenotypes such as rosette formation are thought to be influenced by the ABO blood group, where by O individuals being lower risk of severe malaria while group A are more predisposed individuals to severe malaria (Barragan et al., 2000; Rowe et al., 2007; Vigan-Womas et al., 2012; Goel et al., 2015; Mancio-Silva and Mota, 2015). These are a few examples of the critical roles blood group antigens play in malaria pathogenesis in humans.

In their seminal article, Storry and colleagues showed that SMIM1 protein shares similar biochemical properties with the glycophorins, especially, glycophorin A (GPA) (Storry et al., 2013). These included SMIM1 exhibiting properties of a type I transmembrane protein, possession of possible glycosylation sites and a transmembrane domain with a dimerization motif. This led them to propose a possible function of SMIM1 as a *P. falciparum* invasion mediating receptor. SMIM1 has however been shown to be a type II transmembrane protein (Arnaud et al., 2015). Nonetheless, the protein contains phosphorylation sites within the cytoplasmic region (Arnaud et al., 2015), reminiscent of erythrocyte membrane proteins which have been implicated in *P. falciparum* invasion and development in human erythrocytes (Aniweh et al., 2017). Also, SMIM1 is seen to be phosphorylated in erythrocytes infected with *P. falciparum* schizont stages (Solyakov et al., 2011). *P. falciparum* infection-induced phosphorylation of erythrocyte membrane proteins such as band 3 is known to be critical for parasite egress (Kesely et al., 2016; Pantaleo et al., 2017), possibly due to weakening of the membrane as a result of phosphorylated band 3 dissociating from the cytoskeleton leading to disruption of the membrane's cytoskeletal organization (Ferru et al., 2011). Structurally, it has previously been suggested that the SMIM1 forms part of the same junctional membrane complex with glycophorin C, the Rh antigen and the Kell glycoprotein (Issitt et al., 1994; Daniels, 2013). Given the possible connection between SMIM1 and the structural organization of the erythrocyte membrane, its phosphorylation in *P. falciparum* infected erythrocytes and the similarities shared with the glycophorins, its potential role in *P. falciparum* infection requires further investigations.

The malaria parasite is one of the most successful human parasites, and arguably the most selective force on the evolution of the human genome. Conditions such as sickle cell anemia, glucose-6-phosphate dehydrogenase deficiency, and thalassemia have direct correlations with the incidence of malaria (Fortin et al., 2002; Kwiatkowski, 2005; Parikh and Rosenthal, 2008; Anstee, 2010; Egan, 2018). Notwithstanding, the presence of the malaria parasite impacts the expression and extent of sequence variation of erythrocyte surface receptor genes (Mayer et al., 2004; Kwiatkowski, 2005; Ko et al., 2011; Idaghdour et al., 2012; Network, 2015; Dankwa et al., 2017; Leffler et al., 2017). Notably, the absence of the duffy antigen/chemokine receptor CD234 (DARC) in most Africans is believed to be an evolutionary response to *P. vivax* infection (Chitnis and Miller, 1994; Kwiatkowski, 2005). Similarly, the high rate of polymorphisms in the glycophorins, and other invasion-mediating erythrocyte surface receptors in people living in malaria endemic regions is thought to be an adaptation against parasite assault on the erythrocytes (Mayer et al., 2004; Kwiatkowski, 2005). Given the possible involvement of SMIM1 in malaria pathogenesis, it is likely that this gene is under selection in the sub-Saharan African population and thus may exhibit distinct genetics different from those currently known. A recent study in Kenyan children however did not associate *SMIM1* polymorphisms with severe malaria (Ndila et al., 2018). Nonetheless, this does not rule out SMIM1 being involved in parasite development. Historically, despite their role in malaria pathogenesis being ascertained by molecular and biochemical studies, erythrocyte surface receptors have been under-represented in genome-wide association studies aimed at identifying signatures of protection against malaria (Egan, 2018). The observation by Ndila et al. could mean that (a) *SMIM1* is not involved in malaria pathogenesis; (b) the malaria parasite is not a selectable force on the gene; (c) *SMIM1* is functionally conserved and thus less polymorphic; (d) the gene is diverse such that the contribution of individual haplotypes were masked in the gene pool. Critical examination of *SMIM1* genetics in a heterogeneous population such as that of Africans would provide insight into the possible function and evolution of this gene.

## 7 Conclusion

Currently, there are limited data on Vel prevalence and *SMIM1* genetics in sub-Saharan Africa as well as other populations. The only available data, thus far, were obtained from parts of Europe, Ethiopia and South Africa (Haer-Wigman et al., 2015a, Haer-Wigman et al., 2015b). However, these data are very limited and lack the power to make a valid argument of geo-specific prevalence of the blood group. Given evidence provided herein; including (a) lack of linkage between the reference SNPs (rs1175550G and rs143702418CGCA) in people of black descent in comparison to Caucasians; (b) the possible role of SMIM1 in malaria pathogenesis; and (c) the selective pressure malaria exerts on the human genome, suffice it to hypothesize that SMIM1 expression and its genetics in sub-Saharan Africa may differ from currently available data on this fascinating erythrocyte surface antigen. It is imperative to combine genotypic data on the Vel blood group together with phenotypic data to understand its variability in specific populations. As a protein expressed on the surface of both erythroid precursor cells and mature erythrocytes, it is necessary to understand the extent to which the protein functions in connection with the erythrocyte cytoskeleton as well as membrane associated proteins. In addition, the differential interactions that are affected by the phosphorylation of SMIM1 will be necessary to establish its role on the erythrocyte.
